# Development and content validation of an educational manual for promoting physical activity among older adults in India

**DOI:** 10.1038/s41598-026-37388-z

**Published:** 2026-02-06

**Authors:** Simran Mahatme, Mukesh Kumar Sinha, Syam K. Ravindran, Revati Amin, K. Vaishali

**Affiliations:** 1 Department of Physiotherapy, Manipal College of Health Professions, Manipal Academy of Higher Education, Manipal, Karnataka 576104 India; 2 Department of Clinical Psychology, Manipal College of Health Professions, Manipal Academy of Higher Education, Manipal, Karnataka 576104 India; 3 RCI Registered Clinical psychologist, supervisor, and trainer, Connect & Restore (CORE), Kerala, India; 4 Department of Physiotherapy, Kasturba Medical College Mangalore, Manipal Academy of Higher Education, Manipal, Karnataka 576104 India

**Keywords:** Seniors, Physical activity, Educational manual, Empowerment, Validation, Health promotion, Health care, Health occupations, Risk factors, Signs and symptoms

## Abstract

To better address the increasing problem of physical inactivity among the elderly in India, we aimed to determine the attitudes of older adults toward physical activity and to create an educational resource that would encourage them to engage in physical activity. Thus, our goal was to develop an educational manual about physical activity tailored to the older adult population based upon their views about this topic. The manual was created over two separate stages. In the first stage, we evaluated the attitudes, facilitators, and barriers to physical activity among older adults through in-depth telephone interviews. Each of the 13 participants (five female and eight male), interviewed separately for 60 min, completed a semi-structured interview. Thematic analysis of the transcript of the interviews identified four superordinate themes: benefits of physical activity; perceived barriers; factors which motivate older adults to participate in physical activity; and domains of physical activity. These themes allowed us to tailor the manual to the specific needs of older adults, thereby providing us with valuable information from the perspective of older adults about physical activity. In the second stage of the study, we created a culturally congruent physical activity educational manual for use within the Indian population. We then performed content validation on the manual. For content validation, we used a panel of six experts and ten members of the target population to evaluate the relevance and effectiveness of the manual. All of the contents of the manual were ranked as relevant, with 100% relevance reported by the expert panel and 98% relevance reported by the target population. Independent I-CVIs > 0.79 (S-CVI/Ave = 79%) were calculated for each item, indicating that each item of the manual was relevant. An Flesch ease reading score of 80.9 was calculated for the manual, which indicates that it can be easily read by a sixth grade student. The current study provides a contribution to advances in science related to promoting health, by creating a validated, self-explaining, culturally-congruent physical activity educational manual for physically active older adults.

## Introduction

Older adults (i.e., people aged 65 + years) are one of the fastest growing population segments worldwide^1^; and in terms of their implications for public health systems, an increase in age is linked to an increased burden of chronic diseases, decreased functional abilities and increased dependency. India has an older adult population of over 100 million, and this number continues to grow rapidly, thus creating a national concern around promoting healthy aging^2,3^. Promoting physical activity in later life is therefore essential for preserving functional ability, preventing disability, and supporting independent living^4^.

Physical inactivity among older adults is a major public health issue and is strongly associated with chronic disease, reduced mobility, loss of autonomy, and premature mortality^5,6^. In contrast, the promotion of physical activity is widely accepted as a very effective, non-pharmacological strategy for promoting health in later life^7^. Among older adults, consistent evidence exists demonstrating that engagement in physical activity will result in improvements in cardiovascular fitness, maintenance of musculoskeletal strength, reductions in the risk of falls, and enhancements in mental well-being^4,8^. Besides physical benefits, PA contributes to emotional stability, improved cognition, and overall life satisfaction^9^.

The aging process involves multiple domains (biological, psychological, and social), and quality of life is now recognized as a primary outcome of healthy aging^10^. Maintaining independence, functional capacity, emotional well-being, and social engagement are fundamental components of successful ageing. Social interactions through participation in leisure and recreational activities (e.g., visiting with friends, running errands, pursuing hobbies) strengthen social networks, provide emotional support, and are two critical factors that determine life satisfaction in older age. Additionally, recent research suggests that social interaction and leisure participation are each independently predictive of better mental health and quality of life among older adults by providing opportunities for developing emotional resilience and supportive social connections^11^.

Although the health benefits of physical activity are well-documented, participation levels among older adults remain low. Older adults are likely to adopt sedentary lifestyles as they age secondary to decreasing motivation, fear of injury, underlying health conditions, and lack of access to tailor-made exercise programs^12,13^. Furthermore, inadequate information on how to safely engage in exercise and lack of guidance from qualified professionals, and an environment that does not facilitate safe exercise can further contribute to inactivity in older adults^14,15^. Consequently, education-based strategies that emphasize personal relevance, enjoyment, and feasibility are necessary to encourage continued engagement. Exercise programs must therefore be individualized and adapted to personal capability, environmental context, and cultural expectations to achieve sustained behavior change^7,16^.

Educational initiatives are an important factor in changing physical activity behavior among older adults, especially when the educational content addresses the needs and context of the target population^17^. Awareness of physical activity benefits is consistently predictive of adherence to healthy behaviors. Educational materials prepared by healthcare professionals have been found to enhance participation in exercise, self-efficacy, and preventive health behaviors among older adults^2,18^. However, educational manuals developed for commercial purposes are largely developed for western populations, do not consider cultural beliefs, language barriers, and the everyday lifestyle practices and social realities experienced by older adults in India. Therefore, educational interventions that are not relevant to the local context may be less acceptable and less effective.

In addition, older adults gain more value from educational programs when they are involved in the program development process and when the intervention reflects their preferences, experiences, and daily struggles. Engagement-focused approaches enhance acceptance and long-term adherence. Internationally, evidence supports that educational materials are more likely to positively influence health behavior when systematically developed and validated for content^18^.

Regular physical activity is one of the most effective non-pharmacological interventions for maintaining physical functioning and enhancing life satisfaction in older age^19^. Integrating regular PA into daily routines therefore represents a core strategy for promoting active and healthy ageing.

As a result of the increasing incidence of physical inactivity and the culturally specific and socially diverse nature of aging in India, there remains a critical need for the development of culture and population-specific educational resources addressing both physical and psychosocial aspects of health. As a result, this study is directed at developing and validating an educational manual specifically designed to promote physical activity among older adults in India with the ultimate goal of enabling older adults to adopt and sustain physically active lifestyles and to ultimately enhance their quality of life.

### Methods

This project involved the construction and validation of a physical activity educational manual for promoting physical activity among older adults. To ensure that the booklet was grounded in the lived experiences and preferences of older adults, we conducted the study in two phases.

### Phase 1: assessing the needs of the target population through in-depth, semi-structured interviews

We assessed the needs of the target population using semi-structured in-depth interviews. We conducted semi-structured in-depth interviews via telephone were conducted using the interview guide amongst 13 older adults. The interview guide was developed following a review of relevant literature and aligned with the study objectives. Prior to data collection, senior faculty reviewed the guide to ensure content relevance, clarity, and contextual appropriateness. We selected participants using convenience sampling from the community. Insights generated from these interviews using thematic analysis were intended not only to describe attitudes and behaviours related to physical activity but also to directly inform the subsequent development of the educational manual. The sample comprised 13 healthy individuals (5 females, 8 males) with a mean (SD) age of 68 ± 2.7 years. In terms of occupation, the older adults were either retired, self-employed, or homemakers. We included individuals who were able to read, communicate, and be willing to participate in the study. We excluded those with severe cognitive impairment, unstable medical illness, or an inability to participate in a telephone interview. Most of the older adults were quite physically active, pursuing their hobbies. After a brief description of the study, most showed keen interest in reading a booklet about physical activity and consented to participate. The interviews lasted for about 45–60 min and garnered knowledge of the attitudes and behaviors of the elderly toward physical activity. We recorded all interviews using an in-built call recording software of Huawei, Honor 9 Lite Android Smartphone (Model LLD-AL10) after obtaining verbal informed consent.

We manually transcribed the recordings in Microsoft Word 2016 and thoroughly reviewed the transcripts. (changed to active voice) We highlighted relevant statements and manually coded them using Microsoft Excel 2016. We then condensed codes into sub-themes, themes, and superordinate themes through abstraction and generated results based on the final abstraction of superordinate themes. Experts reviewed the analysed data, and we incorporated their recommendations into the development of the booklet. To enhance reliability, two investigators independently coded and resolved documents through discussion until consensus was reached. Insights from Phase 1 were used not only to describe attitudes and behaviours but also to directly inform the subsequent development of the educational manual.

### Phase 2: development and validation of the manual

#### Development of the manual

##### Bibliographic survey

We conducted an independent literature search on physical activity educational materials was in December 2019 and further revised in December 2024 through databases such as PubMed, CINAHL, and Scopus. Evidence identified through the literature review, together with themes emerging from Phase 1 interviews, guided the selection and organisation of content included in the manual. We screened titles and abstracts, then selected relevant articles for detailed reading and data synthesis to structure the contents of the booklet. We included studies if they focused on physical activity promotion or educational materials for older adults and were published in English. We excluded studies involving acute clinical populations or specialized rehabilitation protocols not applicable to community-dwelling older individuals. Evidence indicated that an educational pamphlet was effective in actively engaging the elderly in physical activity.

#### Construction of the manual

We took assistance from an expert in Fine Arts who worked to create illustrations and the layout of the manual so that it would meet the users’ identified needs. We involved a professional graphic designer to provide the visual layout and illustrations to ensure that the graphics provided a clear illustration to assist in clarifying technical terms and to assist in providing access to older adults with different literacy levels. We created a first draft of the Educational Manual that contained the required information, graphics and text. The Educational Manual primarily focused on the pictorial representation of contents with the adaptation of technical terminologies to make it more accessible and readable to the target audience, regardless of their educational status. We used the Flesch Easy Read score to assess the readability of the text^20^. An Flesch ease reading score of 80.9 was calculated for the manual, which indicates that it can be easily read by a sixth-grade student. Readability was assessed using the Flesch Reading Ease Score, which determines the difficulty of text through analysis of the number of syllables in each word and the average number of words per sentence.

#### Validation of the manual

##### Content validation by experts

We evaluated our manually-developed manual through a systematic validation process, using an expert-panel review to evaluate content relevance, clarity and usability. A draft of the educational manual, along with a cover letter, and a pilot-tested critical appraisal sheet were mailed to six subject matter experts in physiotherapy, exercise science, nutrition science, psychology and geriatric health care. Experts were mailed (by email) the manual, a cover letter, and the validation form; they had been provided clear instructions by the cover letter regarding how to measure each item in the validation form. We assessed content validity using empirical techniques that included calculating the content validity index (CVI)^18^. We calculated the Item-CVI (I-CVI), which was computed by the rating of “relevance” for each item divided by the total number of experts. We retained items with I-CVI > 0.79, revised items with I-CVI between 0.70 and 0.79 and removed items with I-CVI < 0.70. We used Microsoft Excel to summarize expert ratings. We calculated Scale-level CVI using the average method (S-CVI/Ave), defined as the mean of all I-CVIs, and the universal agreement method (S-CVI/UA), defined as the proportion of items achieving unanimous expert agreement (I-CVI = 1.00).

To evaluate the relevance of the items, we developed a critical appraisal sheet with the following domains of inquiry: relevance of “each item” in the manual and expert comments for improvement of items in the educational booklet. Experts rated each item on a 4-point Likert scale (“1 *= irrelevant”*, “2 *= somewhat relevant”*, “3 *= relevant”* and “4 *= highly relevant”)*. We considered ratings of 3 and 4 were considered to be valid for the calculation of CVI. Additional recommendations and feedback from experts were collected in the critical appraisal sheet furnished with the cover letter and the manual. Based on expert feedback, all the suggestions were organized, analyzed, and accordingly adhered to. Conflicting feedback was resolved through discussion among the research team. Where suggestions differed in clinical emphasis, decisions were made based on evidence strength and feasibility for older adults.

#### Face validation by target audience

After expert validation and revision, we evaluated the refined version of the manual was subsequently evaluated by the target audience to assess clarity, acceptability, and cultural appropriateness. We disseminated the revised version of this manual among a small group of 10 target audiences for face validation through email. Participants aged sixty-five and above, literate, able to read the manual, and willing to participate were eligible for the validation process. Participants represented both genders and varied educational backgrounds. We developed a critical appraisal sheet with five domains and 14 items to capture the participants’ opinions about the cover page and title, domains and subdomains, contents, writing, illustration, and cultural adaptation. Participants reviewed the booklet and rated each item for acceptance and relevance (Not relevant, somewhat relevant, relevant, and very relevant). Older adults were asked to review the booklet and analyze texts and illustrations. For individuals who did not understand the written text, we provided one-to-one online sessions for additional assistance. We analysed data from expert and face validation descriptively and calculated CVI for each item, keeping cut-off score equal to or greater than 0.79.

#### Ethical aspects

We conducted this study in compliance with ethical standards and regulations to ensure the safety and rights of participants. Approval was granted by the Institutional Ethics Committee of Kasturba Hospital, Manipal, Karnataka, under the protocol number IEC No. 1025/2019.In addition, we registered this study with the Clinical Trials Registry - India (CTRI) CTRI/2020/03/024310. We screened all volunteers for eligibility, and informed consent was obtained prior to participation. We informed participants that their involvement was entirely voluntary and that they could withdraw from the study at any point without any penalty or impact on their access to healthcare services. We maintained confidentiality and anonymity throughout the study; we did not record any identifiable information in transcripts or reports, and we stored all data securely with access restricted to authorised research personnel only.

## Results

**Fig. 1:**
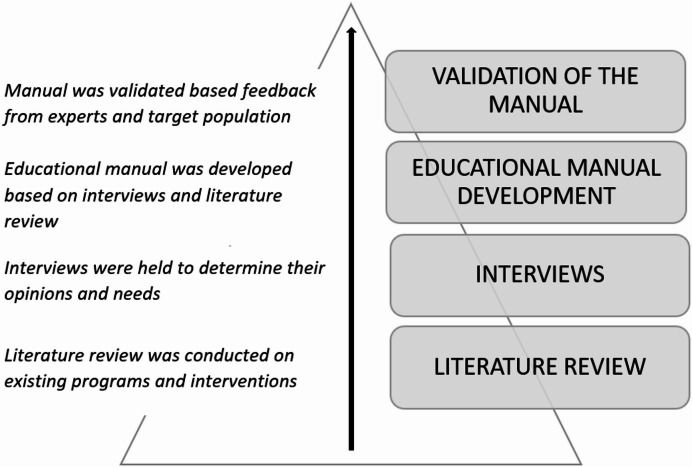
Summary of the process of development of the educational manual “fit4function.

The educational manual “fit4function” was developed through an initial coding process that involved transcription of data obtained from the in-depth interview, resulting in 94 codes. These codes were classified into nine emergent themes. These themes were further subsumed under four superordinate themes (Table 1) that serve as the overarching categories under which the aforementioned themes are better understood. A summary of the process of development of the educational manual “fit4function” is depicted in Fig. 1.

**Table 1:** Superordinate themes.

Number	Superordinate Themes
1	**Benefits of Physical Activity**
1.1	Psychological benefits
1.2	Physiological benefits
2	**Perceived Barriers**
2.1	Psychological barriers
2.2	Physical barriers
2.3	External barriers
3	**Motivators to Physical Activity**
3.1	Intrinsic Motivators
3.2	Extrinsic Motivators
4	**Domains of Physical activity**
4.1	Intentional
4.2	Incidental

Subsequent analysis was undertaken to derive conclusions from the same. These codes aided in identifying the older adults’ current perspective and attitude towards physical activity, which led to the development of an all-inclusive educational manual.

Three physiotherapists, one exercise scientist, one psychologist, and one geriatric healthcare provider comprised the expert panel for content validation of the manual. In terms of professional profile, three experts had a doctoral degree, and three had master’s degrees. All experts were professors with experience in writing educational material. The mean time of expertise in health education was about ten years (SD = 6.9), and twelve years in geriatric health (SD = 2.0). A target population with a minimum age of sixty-five years and a maximum age of eighty-five years was identified as eligible, with a mean of 6 years. Regarding the level of education, older adults who could read, write, and communicate were included.

The preliminary version of the educational manual consisted of ten double-sided pages measuring 210 × 227 mm. Older adults testified that the cover illustration and title description stimulated interest in reading the educational manual. However, the judges suggested an improvement in the depiction of the illustration on the cover page and title. The changes were accepted to improve the quality of the cover. The manual underwent two rounds of revisions.

The proportion of relevance (S-CVI/Ave) of the first version (Table 2) of the manual was 79% among the six judges. An S-CVI/Ave value of 0.79 suggested that the manual needed revisions. The I-CVI rated separately for each item ranged from 0.5 to 1.0. Of the 24 items, 19 (79%) were considered relevant. Sixteen items had a CVI of 0.83, and 3 had a CVI of 1.0. Thus, most items were considered relevant except for five items. These items constituted the title, writing style in the manual, the vocabulary used, two illustrations that the authors found challenging to understand, and the presentation sequence. CVI results have been presented as follows:

**Table 2:** CVI (first revision).

Items	Experts						I-CVI
	1	2	3	4	5	6	
Meets the goals of the elderly and helps them understand physical activity	1	1	1	0	1	1	0.83
Adequate for use by older adults and healthcare professionals	1	1	1	1	1	1	1.0
The cover Page contemplates the necessary information	1	1	1	1	1	1	1.0
The title and topic content are suitable	1	0	1	0	1	1	0.6
There is consistency between the cover, contents, and presentation of the manual	1	1	1	0	1	1	0.83
The number of pages is Adequate	1	1	1	0	1	1	0.83
Manual is of adequate size, neither too small, nor too extensive.	1	1	1	0	1	1	0.83
Writing of the manual and text	1	0	1	0	1	1	0.66
Vocabulary is simple	1	1	0	0	0	1	0.5
Concepts are addressed clearly	1	1	1	0	1	1	0.83
Theme and corresponding text are associated	1	1	1	0	1	1	0.83
The visual composition of the manual	1	1	1	0	1	1	0.83
Organization of the pages	1	1	1	0	1	1	0.83
The number of figures is sufficient	1	1	1	1	1	1	1.0
The size of the figure is appropriate	1	1	1	0	1	1	0.83
Figures are simple, appropriate, and easily understood	1	1	1	0	1	1	0.83
Figures are self-explanatory	1	1	0	0	1	1	0.66
The manual is appropriate for age, gender, and culture	1	1	1	0	1	1	0.83
The manual presents a logical sequence of physical activity	1	1	1	0	1	0	0.66
The manual stimulates interest and curiosity	1	1	1	0	1	1	0.83
The manual promotes behavior change and attitude	1	1	1	0	1	1	0.83
The content of the manual maintains the dynamics of reading	1	1	1	0	1	1	0.83
The content of the manual arouses motivation to read till the end	1	1	1	0	1	1	0.83
The use of this manual serves the purpose and becomes relevant	1	1	1	0	1	1	0.83
**S-CVI/Ave**							**0.79**

The second round of CVI was conducted for the revised manual after incorporating expert recommendations. The revised manual was built with 10 double-sided pages, including a cover page measuring 210 × 227 mm. The first two pages presented text and graphical displays on aging and the importance of physical activity. Page three illustrates participants’ perceived barriers and strategies to overcome them. The concept of personal safety during exercise is introduced via texts and illustrations on pages four and five. On pages six and seven, illustrations depicted the warmup exercises that could be performed before beginning an exercise program with an introduction to four different physical activity concepts. Page eight enlightened on the different aerobic activities that older adults could perform. Pages nine, ten, and eleven showed graphics and texts on upper and lower body strength training for the elderly. The twelfth and thirteenth pages were about flexibility. From the fourteenth to the seventeenth page, the illustrations and texts depicted static and dynamic balance training in participants, both with and without support. The last pages emphasized cool down and special considerations to exercise.

The revised manual draft was circulated among the same panel of experts. However, two experts could not participate in the validation process due to personal commitments. Thus, the manual was validated by a panel of 4 experts. The proportion of relevance (S-CVI/Ave) of the revised version (Table 3) of the manual was 100%, with an S-CVI/Ave value of 1.0.

**Table 3:** CVI (second revision).

Items	Experts				I-CVI
	1	2	3	4	
Meets the goals of the elderly and helps them understand physical activity	1	1	1	1	1
Adequate for use by older adults and healthcare professionals	1	1	1	1	1
The cover Page contemplates the necessary information	1	1	1	1	1
The title and topic content are suitable	1	1	1	1	1
There is consistency between the cover, contents, and presentation of the manual	1	1	1	1	1
The number of pages is Adequate	1	1	1	1	1
The manual is of adequate size, neither too small nor too extensive.	1	1	1	1	1
Writing of the manual and text	1	1	1	1	1
Vocabulary is simple	1	1	1	1	1
Concepts are addressed clearly	1	1	1	1	1
Theme and corresponding text are associated	1	1	1	1	1
The visual composition of the manual	1	1	1	1	1
Organization of the pages	1	1	1	1	1
The number of figures is sufficient	1	1	1	1	1
The size of the figure is appropriate	1	1	1	1	1
Figures are simple, appropriate, and easily understood	1	1	1	1	1
Figures are self-explanatory	1	1	1	1	1
The manual is appropriate for age, gender, and culture	1	1	1	1	1
The manual presents a logical sequence to physical activity	1	1	1	1	1
The manual stimulates interest and curiosity	1	1	1	1	1
The manual promotes behavior change and attitude	1	1	1	1	1
The content of the manual maintains the dynamics of reading	1	1	1	1	1
The content of the manual arouses motivation to read till the end	1	1	1	1	1
The use of this manual serves the purpose and becomes relevant	1	1	1	1	1
**S-CVI/Ave**					1.0

All the items were considered relevant. However, a few changes were recommended. Table 4 highlights the expert comments in the second phase of content validation.

**Table 4:** Expert recommendations.

Page Number	Recommendations
Cover	
1	“darken the images” “change feeling sad to mood/memory disorders” “stick to diagrams that exercise can help”
3	“reduce the size of the two images and increase the font size of the statements” “space out the text” “try showing a physio instead of medical person, instead of steth may be a physio working with old age person”
4	“check for grammar, difficulty breathing and fast beating heart” “reduce size of doctors image and question box” “maintain uniformity of font size” “looks haphazard, rearrange the contents”
6	“increase font size for headings of each exercise” “in old age every movement will cause pain and discomfort, this is not required”
7	“specify time duration of food intake, before activity”
8	“specify how much more clearly” “reduce font size for “how much box”, according to the text”
9	“one arm row diagram not clear” “put arrow over shoulder shrugs”
10	“state how much at either the beginning or end of strength” “reduce font size for “how much box”, according to the text” “show arrow for knee curls”
11	“check for an alternative word for crunch”
12	“put overall cross mark on flexibility and not on individual stretches” “reduce font size for “how much box”, according to the text” “show only one cross mark at the title, indicating considerations”
13	“exercise for hamstring stretch looks confusing, might need modification” “give description for hamstring stretch, difficult to understand” “remove cross marks, cross marks on the pic looks like don’t do”
14	“rearrange illustration, lighter shade for starting position” “space out distance between the phrases written in green, remove numbers, reduce the font size for the box”
16	“add more pictures in cooldown. Lot of empty space” “2 to 5 min not clear, put it in simpler way” “remove refer board”
17	“specify in clear terms on medication” “reduce size of the circles”
18	“increase font size, reduce distance between images”
19	“The arrow looks big, bold and weird. Please change the colour to grey and make it a little smaller” “the contents of the badge/bracelet and description are very small”
General comments	

The recommended changes were incorporated and mailed to 10 target audiences for face validation. A face validation sheet with 14 items was developed using a 4-point Likert scale (not relevant, somewhat relevant, relevant, very relevant) to review the educational manual. The items addressed older adults’ opinions on adequacy, title and cover, text and vocabulary, illustrations, motivation to read and cultural congruency. Additional space for comments and suggestions was provided at the end of the sheet. Table 5 below provides evaluation of manual by the target audience, according to the relevance.

**Table 5:** CVI (face validation).

Items	Target Audience										I-CVI
	1	2	3	4	5	6	7	8	9	10	
Makes it easy to understand physical activity	1	1	1	1	1	1	1	1	1	1	1
Adequate for use	1	1	1	1	1	1	1	1	1	1	1
Cover page	1	1	1	1	1	1	1	1	1	1	1
Title and topic	1	1	1	1	1	1	1	1	1	1	1
Content’s presentation of the booklet is uniform	1	1	1	1	1	1	1	1	1	1	1
Writing in the booklet	1	1	1	1	1	1	1	1	1	1	1
Language is simple (in common words)	1	1	1	1	1	1	1	1	1	1	1
Image and corresponding text are associated	1	1	1	1	1	1	1	1	1	1	1
Overall booklet appears attractive	0	1	1	1	1	1	1	1	1	1	0.9
Pages are well organised	1	1	1	1	1	1	1	1	1	1	1
Size of the figure is appropriate	1	1	1	1	1	1	1	0	1	1	0.9
The booklet is appropriate for age, gender and culture	1	1	1	1	1	1	1	1	1	1	1
Does the booklet motivate you to exercise?	1	1	1	1	1	1	1	1	1	1	1
The content is appropriate and is to understand by local people.	1	1	1	1	1	1	1	1	1	1	1
**S-CVI/Ave**											**0.98**

The proportion of relevance (S-CVI/Ave) of the 14 items was 98% among the target audience. Twelve items received an I-CVI of 1.0. Two items obtained an I-CVI of 0.9 based on the size of the figures and the attractiveness of the overall manual. The I-CVI rated independently for each item > 0.79 which suggested that all items were relevant. Table 6 highlights the recommendations by the target audiences.

**Table 6:** Expert recommendations.

Page Number	Recommendations
1	“figure could have been more colorful.”
6	“font is not clearly visible,” “provide a description of the exercise”
9	“figures can be big.”
General Comments	“A video link for each exercise could be added to increase engagement and facilitate better understanding” “Pictures are good but put a little bit of writing about the exercise” “The size of the figure can be increased”

Most recommendations were based on illustrations and descriptions on pages 1, 6, and 9 of the manual. General recommendations involved providing descriptions of the activities and increasing the size of illustrations. Another unique suggestion that we received was to incorporate video links to specific exercises that could be referred to in case of doubts. The manual also received positive comments in terms of its presentation and cultural considerations. One of the target validators positively commented, *“It highlights the myths and the stereotypes. It establishes a connection with the reader and sets the tone on how one should approach.”* He also added, *“The cautions (e.g. picture of Safety First) one should take are conveyed before starting regular exercise in full flow*,* which is a positive thing” and “Visuals and steps for each exercise are very helpful.”*

However, after validation from experts and the target audience, the final version of the manual included simpler terminologies and four additional illustrations for a better understanding of concepts. A few pages were re-arranged to follow a logical sequence of presentation. Following the recommendations, modifications were incorporated.

## Discussion

As the literature continues to demonstrate the importance of understanding what motivates older adults to be physically active, the study supported the necessity to know the physical activity views of older adults and the factors that contribute to their willingness to engage in PA. Positive attributes of PA that were noted by participants included improved general well-being, maintenance of independence, and greater social interaction. However, participants identified multiple barriers to consistent physical activity including physical limitations, fear of injury, limited tailored information and motivational barriers. The study’s findings indicate that merely instructing older adults to “exercise” will be insufficient to promote PA without acknowledging their experienced environment and their lived experience. To create the culturally adapted educational manual to accommodate the unique beliefs, fears and needs of the participants, the purpose of the educational manual was created.

Both gerontologists and older adults validated the manual. Validation of the manual indicates that it is appropriate for use in everyday practice and demonstrates the manual’s ability to positively affect the quality of life and physical health of older adults.

While extensive research exists documenting the association of PA and health promotion in older adults, little research has examined the perceptions and attitudes toward PA in older adults. While there is a world-wide increase in PA programs for the prevention of chronic diseases, little research exists regarding physical activity programs specifically for seniors^21^. This indicates a large discrepancy in promoting PA and identifying behavioral drivers of physical activity participation. Since the value of educational materials is reduced if they do not accurately represent the authentic experiences of the target group and do not represent problems perceived by the target group^18^, this study used qualitative methods to explore the behavioral determinants of PA in late adulthood. The semi-structured interview format provided participants with the opportunity to highlight the issues that they believed would assist them in engaging in PA^22^. This format provided participants with more autonomy to discuss topics of interest, thus reducing the risk of researcher-imposed bias.

Participants identified both the positive and negative aspects of PA; however, participants did not identify any strategies to overcome the barriers to PA. Semi-structured interviews elicited more involved responses from participants than structured or quantitative methods, because participants had more control to elaborate on each question^22,23^. These characteristics of the methodologies used added to the credibility of the findings.

PA behavior is affected by physical, psychological, and social determinants^23^. As such, the manual was designed to provide both physical activity instruction and to address fears, misconceptions, and safety concerns associated with physical activity. Contributions to the development of the content were made by experts in gerontology, rehabilitation, and psychology^2,18,26^. A multidisciplinary approach was taken to improve the clinical accuracy of the manual and its cultural sensitivity.

Effectively designed educational materials have the capacity to impact health behaviors^2^, particularly when the materials utilize the language and lifestyle of the target population. It is necessary for the information disseminated to be both relevant and understandable to the target population. Findings from this study indicated that participants desired physical activity recommendations that were simple and easy to understand within the educational material. The manual represents a means of distributing information regarding physical activity to the target population. Illustrations and simplified language within the manual are utilized to provide participants with safety information, making the manual more easily understood by individuals who have varying levels of literacy.

To validate the manual using the most common measure to assess content validity, we assessed item-level CVI (I-CVI)^6,7^. Six experts participated in the first round of validation. The range of the I-CVI was 0.50 to 1 with 79% of the items being found relevant. Based on the recommendations from the experts, five items were modified. Most of the modifications were made to ensure comprehensibility, to avoid misinterpretation and to clarify ambiguity^4,7,8^. Multiple rounds of iterative review strengthened the conceptual clarity of the manual.

After modifications, all four experts in the second round of validation rated the I-CVI as 1.  The manual received a Flesch reading ease score of 80.9, which suggests that the manual is readable at approximately a sixth-grade level^19^. This supports the conclusion that the manual can be disseminated via community based distribution.

Following the proposed revisions, we disseminated the revised version of the manual to the target population. The face validation resulted in an S-CVI/Ave of 0.98. This illustrates high end-user acceptance^26^. Consequently, the manual was validated with a 98% relevance proportion.

Several limitations exist to the current study that must be addressed. The sample size was comprised of 13 participants, limiting the transferability of the findings. Telephone interviews may limit the verbal communication capabilities of the participants and potentially exclude participants with hearing impairments. The study was limited to examining one culture and therefore, the findings may not be applicable to different geographic regions. No assessment of the participants’ PA behavior or functional outcomes was performed.

Further research could evaluate how well the manual is used in clinical trials that are conducted in communities; whether a digital version of the manual would be as effective; and whether or not older adults will continue to engage in physical activities over time. Researchers could also need to conduct studies on implementing the manual in physician offices, physical therapy clinics, and adult day centers for seniors.

## Conclusion

The development and validation of the PA educational manual for older adults in India is significant because it provides a basis for improving geriatric public health practice.

An iterative approach was used to develop the manual with the involvement of the target audience and expert reviewers; therefore, formal validation using expert review and user experience validation was used to evaluate the relevance, clarity and accuracy of the content within the manual.

High content validity and readability scores for the manual are indicative of the manuals’ scientific and community based use. Because PA has been shown to have a positive effect on health and well-being in older adults, the present validated manual serves as a practical and accessible resource for encouraging older adults to safely and consistently engage in PA. The educational manual was developed as a visually enhanced, scientifically based resource to aid in independent exercise in community settings.

### Practice implications

Recommendations for the dissemination of the manual include use in primary care centers, physiotherapy clinics, and through community-based senior welfare programs to assess if physical activity behaviors are changed among participants. Healthcare practitioners and physiotherapists can utilize the manual during routine patient visits and/or during community workshops to emphasize key messages and to provide demonstrations of safe exercises. Incorporating the manual into existing national health promotion and eldercare programs may increase the potential reach and utilization of the program to the population level. Adaptation of the manual into future digital audio/visual versions may also enhance accessibility to the manual for those with low literacy and/or visual impairments.

## Data Availability

Research data will be available from the corresponding author with a reasonable request.
